# Bone Age Assessment Methods: A Critical Review

**DOI:** 10.12669/pjms.301.4295

**Published:** 2014

**Authors:** Arsalan Manzoor Mughal, Nuzhat Hassan, Anwar Ahmed

**Affiliations:** 1 Dr. Arsalan Manzoor Mughal, MBBS, M. Phil Candidate, Senior Lecturer, Department of Anatomy, Ziauddin University, Karachi, Pakistan.; 2 Dr. Nuzhat Hassan, MBBS, M.Phil (Anatomy), Professor & Head of Anatomy Department, Ziauddin University, Karachi, Pakistan.; 3 Dr. Anwar Ahmed, MBBS, FMRD, MD, FCPS, Associate Professor of Diagnostic Imaging Dr. Ziauddin Hospital, North Nazimabad, Karachi, Pakistan.

**Keywords:** Bone Age Measurement, Diagnostic X-Ray Radiology, Ultrasonic Diagnosis, Panoramic Radiography, Clavicle, Ilium, Femur Head

## Abstract

The bone age of a child indicates his/her level of biological and structural maturity better than the chronological age calculated from the date of birth. Radiography of the hand & wrist is the commonest modality used to calculate bone age. Automated methods for evaluation of hand and wrist radiographs are also being developed which reduce inter rater variability compared to manual methods. Non radiation based techniques of visualizing hand & wrist bones such as ultrasonography for bone age calculation have been theorized but are not as accurate as radiographic methods. By the age of 18 years, bone age cannot be computed from hand & wrist radiographs, therefore the medial end of the clavicle is used for bone age calculation in individuals aged 18—22 years. CT visualization of the clavicle has been extensively studied but requires a high dose of radiation. MRI based methods are being developed but require more research. Dental age is an alternate form of bone age determination, which also gives an estimate of skeletal maturity. The iliac bone and femoral head have also been studied for computation of bone age but no standardized methods have yet been generated.

As different modalities of bone age estimation provide different results and their applicability differs in different ethnicities, we need to design studies in order to compare them and select the method best suited to Pakistani children.

***Sources of Data/Study Selection:*** Recent articles published between years 2004-2013 obtained from online search engines Pubmed and Google Scholar were used in preparation of this review.

## INTRODUCTION

Bone age is an indicator of the skeletal and biological maturity of an individual. This is different from chronological age, which is calculated using the date of birth of an individual. Bone age is often requested by pediatricians and endocrinologists for comparison with chronological age for diagnosing diseases which result in tall or short stature in children. Serial measurements are also used to assess the effectiveness of treatments for these diseases.^1^ Furmulae have also been designed for computing the final adult height of children from bone age values in normal healthy children.^2^

Calculation of bone age is also employed for estimation of chronological age in conditions were accurate birth records are not available. Absent birth data is a big problem in our part of the world. In South Asia, 65% of all births are not registered by the age of 5 years.^3^ Thus need for accurate estimation of age arises in conditions where the age of a child needs to be accurate, such as during immigration^4^, in law suits^5^ and in competitive sports.^6^ In these cases bone age is used to provide the closest estimate of chronological age.

In order to compute bone age various methods have been developed using different skeletal elements and various visualization techniques. A critical comparison of these methods is given below.

**Bone age by visualization of Hand & Wrist bones**

The pattern of ossification in the hand and wrist bones is in a fairly predictable manner and age specific until end of adolescence when the elongation of bone is complete. Thus, the standards of bone age have been derived by comparing the level of maturation of hand and wrist bones with normal age levels.

Traditionally, the extent of growth and development of hand bones has been visualized by plain wrist radiographs, however newer methods such as ultrasound of hand bones are being tried but have yet not been validated.

***Visualization by plain hand & wrist radiographs: ***There have been great advancements in radiological techniques over the past few decades but to date, plain radiographs of the hand are the investigation of choice for bone age assessment. A standard posterior-anterior (PA) view of the hand and wrist is ideal for visualization of features of hand bones.^7^

The hand radiographs are quite safe to obtain as the effective dose of radiation received during each exposure is between 0.0001-0.1 mSV.^8^ This dose is less than 20 minutes of natural background radiation or the amount of radiation received by an individual on a 2 minutes transatlantic flight.^1^

Various methods have been developed to compute bone age score from these radiographs by comparing the maturity of hand & wrist bones to idealized standards. A brief description of the commonly used methods is given below.

***1: The Greulich & Pyle (GP) Atlas: ***Is a holistic method based on “The *Radiographic Atlas of Skeletal Development of the Hand and Wrist”*, by Dr William Walter Greulich and Dr Sarah Idell Pyle, its last edition published in 1959, is still one of the most commonly used atlas for bone age measurement by radiologists in Pakistan^9^ and in the West.^10^ It contains reference images of male and female standards of the left wrist and hand from birth till 18 years for females and 19 years for males. Also, explanation regarding the gradual age related changes observed in the bone structure is provided with each standard image. Bone age is calculated by comparing the left wrist radiographs of the subject with the nearest matching reference radiographs provided in the atlas which are standard for different ages provided in the atlas.^10^

This method is simpler and faster than other radiograph based methods.^1^ GP atlas standards are considered applicable and reliable for children in Australia^11^ and Middle East.^12^ However, disparity between the calculated bone age and chronological age is noted when this method is applied to Asian children.^9^^,^^13^

***2: Tanner*** ***Whitehouse (TW2) Method: ***The Tanner &Whitehouse (TW) method in contrast is not based on the age, rather it is based on the level of maturity for 20 selected regions of interest (ROI) in specific bones of the wrist and hand in each age population. The development level of each ROI is categorized into specific stages labeled as (A, B, C, D, . . ., I). A numerical score is given to each stage of development for each bone individually. By summing up all these scores from the ROIs, a total maturity score is calculated. This score is correlated with the bone age separately for males and females. TW method is comparatively more complex and requires more time; however it is more accurate and reproducible when compared to GP method.^14^

***3: The Gilsanz & Ratibin (GR) Atlas: ***A new digital atlas developed by Vicente Gilsanz and Osman Ratibin^15^ in 2005. They produced idealized and artificial images specific for age and sex standards of skeletal maturity by thoroughly analyzing the size, shape, morphology and density of ossification centers in hand radiographs of healthy children, and generating images that include the typical characteristics of development for each of the ossification centers.^16^ The images of the new GR atlas are much more precise and have a better quality than those of the older GP atlas.^17^ Also these new GR atlas standards are spaced at regular 6 monthly intervals from the ages of 2 to 6 and at yearly intervals from age 7 to 17.

It has been observed that both pediatric endocrinologists and radiologists showed nearly identical results in determining bone age from GP and GR atlas. However the GR atlas had an increased number of outliers. Still it can be used to replace the older GP atlas.^16^

***4: Automatic Skeletal Bone Age Assessment: ***Manual estimations of bone age by the above mentioned methods do have some degree of inter rater variability. This creates problems in its clinical application, comparison between subjects and follow-up of patients. A computerized automatic system of bone age assessment would in theory be a solution^18^, but practically it is very difficult to generate an automated system that could accurately analyze the variations, size, shape and mineralization in multiple ossification centers in the hand and wrist bones.^15^ Computerized calculation of bone age from wrist radiographs has been around for the past 3 decades. Radiographs are either obtained by digital radiography or digitalized via a scanner and then undergo several steps. During pre-processing the image is normalized to grayscale so that important segments of the image can later be extracted, background is removed and orientation of the image is corrected. In the next step called segmentation, desired portions of the bones and soft tissue are separated from the background. Then the image is analyzed by taking account of selected regions of interest for calculating bone age by Tanner-Whitehouse method or by comparison with standard images for estimation by Greulich & Pyle Atlas.

Older methods of automated Image-processing that detect features of ossification in hand bones showed significant variation from bone age calculated by manual methods. However a newer method for automatic bone age assessment called Bone Xpert has been generated that rebuilds the edges of 15 bones of interest in hand radiographs and uses this information to compute bone age by both the Greulich Pyle (GP) and Tanner Whitehouse (TW) methods.^19^ The use of this software has been validated for various ethnicities.^20^

***Visualization by Ultrasound: ***BonAge is an ultrasound device which includes an ultrasound probe connected to a main unit for calculation of bone age. The method uses two transduces, one that produces Ultrasonic waves with a frequency of 750 kHz directed at the epiphysis of distal end of Ulna and radius whereas the other acts a receiver. The entire process takes about 5 minutes in which eleven cycles of measurement are completed to provide accurate results. A skeletal age is computed using information obtained from demographics of the subject and the ultrasound results.

Bone age calculated using ultrasound is still in initial stages and needs further refinement.^21^ However, initial studies on comparison with the GP atlas standards show promising results.^22^^–^^24^ However in a few studies wide discrepancies are also noted.^25^ In order to replace the wrist radiographs, this method needs to be evaluated in multiethnic population with a large sample size.

***Bone age by visualization of dental maturity: ***Skeletal age calculated by assessing dental maturity has extensively been applied for forensic purposes but literature on its use in diagnosis and follow-up of endocrine diseases is scarce. This is probably because the degree of mineralization of tooth is much less affected by nutrition and endocrine states compared to mineralization in limb bones. However, just like hand bones, two similar approaches are used for computing dental age.

***1: Atlas Method: ***This is a holistic method where stages of mineralization in various teeth are collectively visualized in an orthopantomograph and matched with standard age images in an atlas. The first atlas was developed by Schour et al.^26^ in 1944. Later new atlas was generated by Moorrees et al.^26^ in which distinct stages of dentition of various teeth were specified. Moorrees’s method was further modified by Anderson et al.^26^ where they defined dentition stages for all teeth.

***2: Scoring Method: ***In contrast, this is a numerical method devised by Demirjian et al.^26^ in 1973 in which each tooth is assigned a maturity score based on the level of dentition. A total maturity score is calculated by adding up all the individual maturity scores and used to calculate dental age. This method was found to give a bone age assessment that highly correlated with chronological age in Indian population.^27^^,^^28^ Also no significant differences have been found between age estimations from wrist radiographs by Greulich Pyle Method and bone age estimations from orthopantomographs by Demirijian method and can be used to replace each other.^29^ Recently 3 new methods of scoring (Willems I, Willems II, and Chaillet standards) have been developed and are considered more accurate for French^30^, Spanish^31^, Venezuelan^31^ and Malaysian^32^ children.

***Bone age by visualization of the clavicle: ***Clavicle is the first long bone to start ossifying in fetal life. The ends ossify by endochondral ossification whereas the shaft ossifies via membranous ossification. During adolescence, uniquely, a secondary epiphyseal ossification center appears at the medial end of the clavicle that results in growth and remodeling of the bone till complete fusion occurs at approximately 22 years. By the age of 18 years, hand ossification, third molar mineralization and sexual maturation are complete so hand radiography and dental age assessment are futile. So, imaging of medial end of clavicle is used for calculation of bone age of individuals of ages 18-22 years.

Conventional radiography of the clavicle is often plagued by overlapping shadows produced by structures of mediastinum, the vertebrae and the ribs. This results in inaccurate visualization of the medial epiphysis and thus cannot be used for staging the extent of maturation. Conventional multidirectional tomography can be used but images produced by this method are also not upto the standard. Computed tomography (CT) on the other hand provides more accurate structural features of the clavicle as well as the surrounding soft tissue structures. Spiral CT requires shorter time to perform resulting in better patient compliance and less artifacts, but has a higher radiation dose when compared with standard CT.^33^

Magnetic Resonance Imaging (MRI) of the clavicle is an upcoming method of bone age determination. This technique being radiation free would decrease the amount of radiation exposure caused by standard or spiral CT. But for this purpose, a specific protocol needs to be generated and more research is required.^34^

3 Tesla MRI is superior to 1.5 Tesla imaging as it gives better contrast and signal to noise ratio resulting in more accurate visualization of the tiny cartilage around the medial end of clavicle. There are only two studies available to date to describe the developmental stages of clavicle by MRI imaging, one based on the 1.5 Tesla imaging^34^ and the other based on 3Tesla imaging^35^ making this an area for potential and fruitful research.

***Bone age by visualization of the iliac bone: ***The process of ossification and fusion of iliac bones has been extensively studied by radiography.^36^ The method used is called the Risser sign, which is based on the degree of maturation of the iliac crest apophysis. Literature review suggests that the ossification of iliac crest apophysis is not uniform resulting in discrepancies while using this method for bone age calculation. This is why it is not used as a replacement of bone age calculation from hand radiographs. Newer methods are being developed to compute bone age from iliac radiographs^37^ but further studies are needed to compare different grading systems.

Non-ionizing imaging procedures for visualization of iliac crest are also under research. A pilot study has been conducted on ultrasonographic visualization of ossification in the cartilaginous part of iliac crest (iliac apophysis) for estimation of bone age which shows promising results.^38^ However to validate and standardize this method further studies are warranted.

***Bone age by visualization of the femoral head: ***An alternative approach for skeletal age assessment can be by assessing the depth of the epiphysial cartilage of femoral head which is continuously being ossified, in contrast to visualizing the bony ephiphysial end of femur. When ossification is complete, most of the cartilage is replaced by bone and the remaining cartilage is called as the “hyaline articular cartilage”.

Only one study is available on the ultrasonographic measurement of the thickness of anterior femoral head cartilage with relatively small number of subjects and lack of racial heterogeneity. Limitations of using this method for skeletal age estimation also include involvement of articular cartilage in various juvenile diseases of the hip joint.^39^

## CONCLUSION

Our critical literature review reveals that there is no standard method for bone age assessment. The most commonly used and extensively developed methods use Hand & Wrist radiographs in children under 18 years and computed tomography (CT) images of medial end of clavicle in individuals aged 18-22 years. Also, some methods are considered applicable to children of certain populations whereas they do not conform to the growth pattern of children in other geographic locations.

We suggest that due to great variations in results from various bone age estimation methods, initially, pilot studies should be carried out using various modalities, on normal healthy children. These results should be compared in order to select and/or develop the best methodology that accurately represents the growth pattern and correlates best with chronological age in Pakistani children. Subsequently, large scale researches should be planned to develop national guidelines of bone age assessment for our children.

## Authors Contribution:

AMM conceived, designed and edited the manuscript.

NH reviewed the anatomical content of manuscript.

AA reviewed the radiological content of manuscript.
